# HyperTMO: a trusted multi-omics integration framework based on hypergraph convolutional network for patient classification

**DOI:** 10.1093/bioinformatics/btae159

**Published:** 2024-03-26

**Authors:** Haohua Wang, Kai Lin, Qiang Zhang, Jinlong Shi, Xinyu Song, Jue Wu, Chenghui Zhao, Kunlun He

**Affiliations:** School of Computer Science and Technology, Dalian University of Technology, Dalian, Liaoning 116024, China; School of Computer Science and Technology, Dalian University of Technology, Dalian, Liaoning 116024, China; School of Computer Science and Technology, Dalian University of Technology, Dalian, Liaoning 116024, China; Research Center for Medical Big Data, Medical Innovation Research Division of Chinese PLA General Hospital, Beijing 100039, China; Research Center for Medical Big Data, Medical Innovation Research Division of Chinese PLA General Hospital, Beijing 100039, China; Research Center for Medical Big Data, Medical Innovation Research Division of Chinese PLA General Hospital, Beijing 100039, China; Research Center for Medical Big Data, Medical Innovation Research Division of Chinese PLA General Hospital, Beijing 100039, China; Research Center for Medical Big Data, Medical Innovation Research Division of Chinese PLA General Hospital, Beijing 100039, China

## Abstract

**Motivation:**

The rapid development of high-throughput biomedical technologies can provide researchers with detailed multi-omics data. The multi-omics integrated analysis approach based on machine learning contributes a more comprehensive perspective to human disease research. However, there are still significant challenges in representing single-omics data and integrating multi-omics information.

**Results:**

This article presents HyperTMO, a Trusted Multi-Omics integration framework based on Hypergraph convolutional network for patient classification. HyperTMO constructs hypergraph structures to represent the association between samples in single-omics data, then evidence extraction is performed by hypergraph convolutional network, and multi-omics information is integrated at an evidence level. Last, we experimentally demonstrate that HyperTMO outperforms other state-of-the-art methods in breast cancer subtype classification and Alzheimer’s disease classification tasks using multi-omics data from TCGA (BRCA) and ROSMAP datasets. Importantly, HyperTMO is the first attempt to integrate hypergraph structure, evidence theory, and multi-omics integration for patient classification. Its accurate and robust properties bring great potential for applications in clinical diagnosis.

**Availability and implementation:**

HyperTMO and datasets are publicly available at https://github.com/ippousyuga/HyperTMO

## 1 Introduction

In recent years, high-throughput sequencing technologies have made breakthrough advances in terms of detection speed, accuracy, and application value (Lee and Yoon 2017), which provide a wealth of multi-omics data for disease research. These multi-omics data represent the essential information of the human body from different perspectives (Lightbody *et al*. 2019), and different omics provide complementary biological information, each having its own advantages and limitations (Tsimenidis *et al*. 2022), life processes are dynamically expressed at multiple levels. Research on single-omics data is fundamentally incomprehensive and does not take into account the information expressed at different levels. Therefore, studying a single omics can only uncover a part of biological complexity, but by integrating multi-omics data, a better understanding of biological complexity can be achieved, and a more comprehensive exploration of the expression processes in life science can be conducted (Mobadersany *et al*. 2018, Pinu *et al*. 2019, Krassowski *et al*. 2020, Picard *et al*. 2021).

Looking at the research history in academia, the earliest methods for disease prediction primarily relied on statistical approaches. These methods involved calculating feature values using detection results of substances such as genes and proteins found in human bodily fluids, urine, or tissues. By comparing these values to predefined thresholds, researchers identified corresponding biomarkers of the respective omics and inferred disease traits based on these biomarkers, such as Genome-Wide Association Studies (Miyazawa *et al*. 2023). However, statistical methods used for mining omics-disease associations have clear limitations. First, these methods focus solely on the statistical calculation of individual markers within each omics, overlooking the potential impact of multiple markers with low statistical values on phenotypes. Therefore, the influence of features with low statistical values on phenotypes cannot be ruled out. Second, since biological regulation processes are multilayered and dynamically expressed, research methods that focus exclusively on a single omics inherently lack the capability to account for the impact of hierarchical regulation within that omics.

In summary, it is necessary to employ a multi-omics integrated approach in the study of omics-disease associations to fully leverage the information from high-dimensional data and gain a deeper understanding of biological systems. With the successful development of artificial intelligence techniques in many fields, deep learning as an important approach to artificial intelligence has become popular in biological field (Huang *et al*. 2019). More and more multi-omics integration approach is proposed for disease classification. For example, Chaudhary *et al*. used automated encoders to reconstruct the features of high-dimensional multi-omics data and clustered the samples by the K-Means method, and finally trained a support vector machine (SVM) model to classify and predict liver cancer (Chaudhary *et al*. 2018). Ma *et al*. combined mRNA features with DNA methylation data to form an integrated feature set and used the XGBoost model to classify cancer patients (Ma *et al*. 2020). Li *et al*. proposed the MoGCN model to merge features and construct a patient similarity network for multi-omics data by using similarity network fusion (SNF) methods, then trained a GCN for cancer subtype classification (Li et al. 2022).

These methods fuse multi-omics data before input to the prediction model is called early integration (Picard *et al*. 2021). Early integration approaches made the model challenging in terms of generalizability and robustness (Reel *et al*. 2021), because the high-dimensional matrix generated by integration is difficult to be analyzed by most machine learning models, and the heterogeneous nature among multi-omics data is also a major factor affecting the model performance.

Late integration is another approach that can be flexibly adapted to different omics data and has received a lot of attention from researchers recently. Islam *et al*. built a deep convolutional neural network to extract features for Copy Number Variation (CNV) data and DNA expression data, and the feature results of each omics data type were input to the connected layer for BRCA subtype classification (2020). Sun *et al*. built independent deep neural network (DNN) model for each omics data type and then a decision level multimodal fusion was used to integrate the multi-omics information (2018). Recently, Wang *et al*. introduced the MOGONET to construct graph structures for each omics data type, graph convolutional networks (GCN) (Kipf and Welling 2016) were used for omics-specific learning, and output results were integrated by View Correlation Discovery Network (VCDN) (Wang *et al*. 2019) to explore cross-omics correlation, the capability of MOGONET for biomedical classification tasks were verified by multiple sets of experiments (Wang *et al*. 2021). The late integration approach analyzes the features of the single-omics data type, and each omics feature was integrated by connection methods. However, recent researches still replicating traditional models (Sun *et al*. 2018), both in the omics-specific learning and the multi-omics integration, leaving the knowledge contained in high-dimensional omics data partially neglected. With the continuous development of deep learning, breakthroughs in disease classification tasks have become possible and significant.

**Figure 1.**
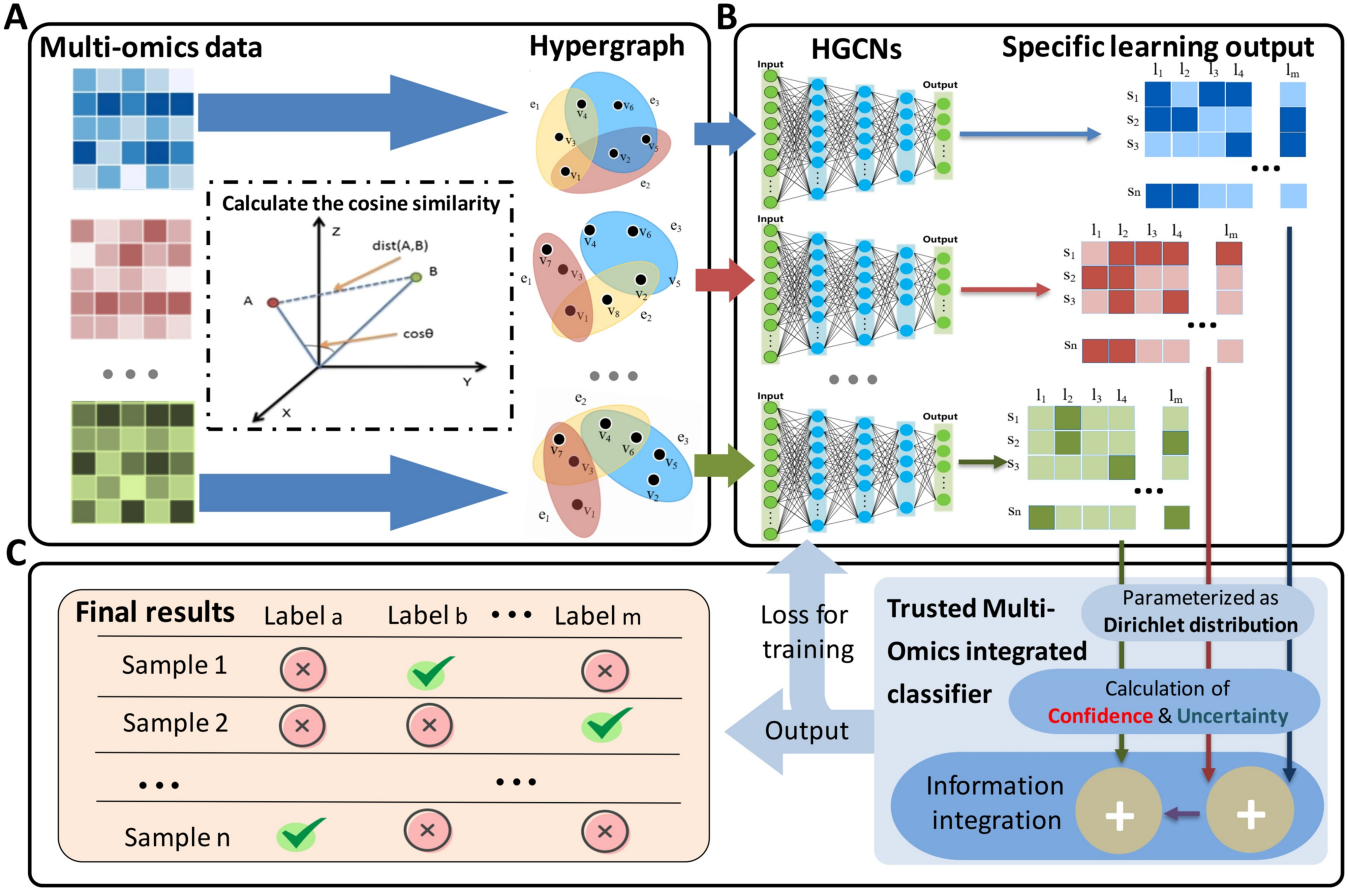
General conceptual framework of HyperTMO. (A) The omics data is used to calculate the cosine similarity for each sample, and then the samples are clustered based on KNN algorithm to get the incidence matrix and form the corresponding hypergraph structure. (B) A hypergraph convolutional neural network (HGCN) is constructed for each omics data type to perform specific learning and output as evidence for multi-omics integration. (C) Dirichlet distribution was parameterized based on the above evidence results, and then the confidence and uncertainty parameters were calculated for each omics data type. Finally, cross-omics information integration was performed based on the D-S evidence theory, and output the classification results with trusted multi-omics integration.

In this article, HyperTMO is proposed to be used for disease classification based on the late integration. We innovate in the single-omics data analysis and multi-omics integration modules, respectively (see Fig. 1 for an overview). Specifically, the single-omics data are used to calculate the cosine similarity for each sample, and the KNN algorithm is used for sample clustering to construct the hypergraph structure. The hypergraph can better represent the underlying relationships than the graph structure. HyperTMO also utilizes hypergraph convolutional network (HGCN) to learn the high-order associations of the omics data. Finally, trusted multi-omics integration classification are performed at the evidence level to solve the information uncertainty problem among each omics data type. HyperTMO is the first disease patient classification framework to combine hypergraph representation with the multi-omics integration method, and the uncertainty estimation multi-omics integration method is also utilized for the first time in the disease classification task. We also validated that HyperTMO outperformed other existing methods in classification performance with multi-omics data from The Cancer Genome Atlas Program (TCGA) (BRCA) and Religious Orders Study/Memory and Aging Project (ROSMAP). In addition, we demonstrate the enhancement brought by the hypergraph structure compared with the graph structure, the anti-noise property of the HyperTMO, and the validity of the trusted multi-omics integration method through comprehensive ablation studies.

## 2 Materials and methods

HyperTMO consists of three components: hypergraph representation, HGCN, and trusted multi-omics integrated classifier. The hypergraph representation module calculates the cosine similarity of different samples within single-omics data and uses the KNN algorithm to construct the incidence matrix of the hypergraph. The HGCN module extracts classification evidence for each omics data type. The trusted multi-omics integrated classifier module utilizes the evidence results output from the HGCNs to parameterize the Dirichlet distribution, and then the confidence and uncertainty parameters were calculated for each omics data type. Finally, cross-omics information integration was performed based on the D-S evidence theory, and made the final classification decision at an evidence level.

### 2.1 Data preparation

To demonstrate the effectiveness of HyperTMO, we performed the BRCA PAM50 subtype classification task and the Alzheimer’s disease classification task using two datasets, including BRCAmulti-omics data from TCGA (https://gdac.broadinstitute.org/) and Alzheimer’s disease multi-omics data from ROSMAP (https://adknowledgeportal.synapse.org/), both containing mRNA data and miRNA data for transcriptomics and DNA methylation data for epigenomics. The BRCA classification task predicts five subtypes: Normal-like, Basal-like, human epidermal growth factor receptor 2 (HER2)-enriched, Luminal A, and Luminal B (Parker *et al*. 2009). Alzheimer’s disease involves the normal control group and the disease group.

It is necessary to compare fairly the performance of HyperTMO with baselines. Therefore, we performed the preprocessing method from Wang *et al*. in MOGONET to remove artifacts and redundant features in each omics data, and MOGONET was compared as a state-of-the-art (SOTA) model in the experimental part. Details of the preprocessing are given in Supplementary Text S1.

To demonstrate the validity of the proposed HyperTMO, ROSMAP, and TCGA (BRCA) datasets were used for the biomedical classification tasks. We compare the performance of HyperTMO with existing classification methods for multi-omics data and conduct comprehensive ablation experiments to demonstrate the necessity of different modules in HyperTMO. We used accuracy (ACC), F1 score, and area under the receiver operating characteristic curve (AUC) to evaluate the performance of the binary classification tasks, and accuracy (ACC), average F1 score weighted by support (F1_weighted), and macro-averaged F1 score (F1_macro) for the multiclass classification tasks. In addition, we use a 5-fold cross-validation scheme where 80% of the data as the training set and the other 20% as the test set. Various methods were evaluated on five training and test sets split randomly at the above scale, and the mean and standard deviation across the five experiments were reported.

### 2.2 Hypergraph construction for omics data

High-dimensional omics data are now commonplace in disease research. Many researchers construct graph structures for omics data and utilize GCN for representation learning (Liu *et al*. 2020, Wang *et al*. 2020, 2021, Li *et al*. 2022, Yin *et al*. 2022). The efficiency of representation learning is directly related to the representation structure, different representation methods determine the performance of the model (Bengio *et al*. 2013). Graph theory cannot precisely represent the underlying knowledge among the features of omics data, because the concept of edges can only model pairwise binary relationships. In addition, this lack of knowledge is amplified when multi-omics information is integrated, leading to limited accuracy and robustness of the model. To overcome the problem, this article constructs a hypergraph structure for each omics data, which can better represent the complex association and heterogeneous of multi-omics data.

**Figure 2.**
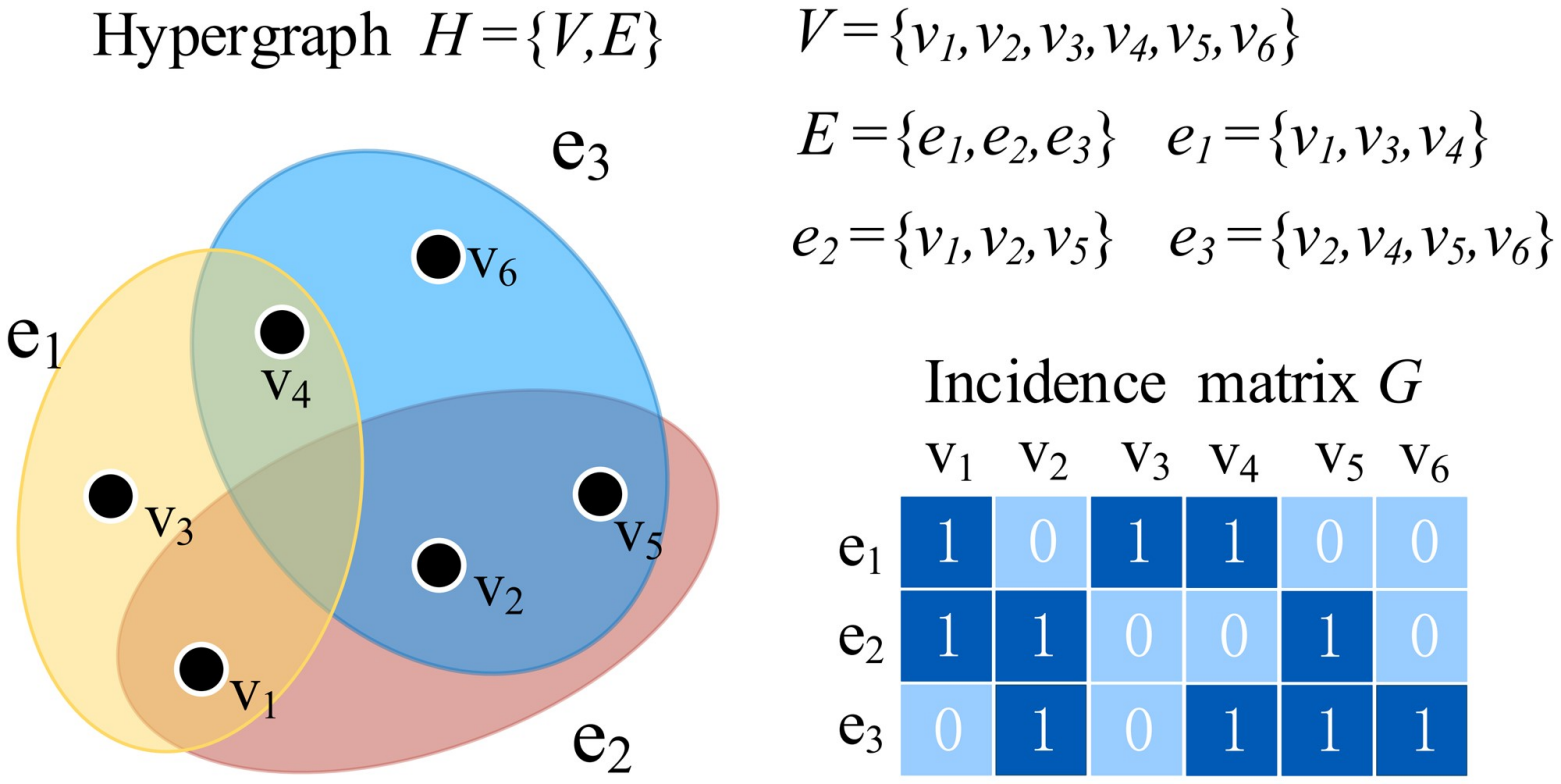
An example of a hypergraph. A hyperedge in a hypergraph connects two or more vertices, which significantly enhances the ability to represent omics data compared to the graph structure.

The hypergraph is a graph structure in a broad sense that breaks the relationship constraint of one edge measuring two vertices. A hyperedge is designed to accommodate several vertices so that the simple graph can be considered as a special hypergraph structure (Gao *et al*. 2020). A hypergraph *H* (Fig. 2) can be defined as H=(V,E). $V=\left\{v_{1},v_{2},\ldots ,v_{n}\right\}$ represents the set of all vertices. Each element of the hyperedge set $E=\left\{e_{1},e_{2},\ldots ,e_{m}\right\}$ contains several vertices. In this article, each vertex corresponds to a patient sample, and each hyperedge contains an arbitrary subset of the set *V* (Lin *et al*. 2022).

Traditional methods usually calculate the Euclidean distance to measure the degree of association between vertices, and connect *k* nearest neighbors for each vertex to construct the hyperedge (Feng *et al*. 2019):

(1) $$\begin{array}{c} d(x_{i},x_{j})=\sqrt{\sum_{r=1}^{R}{\left(x_{ir}-x_{jr}\right)}^{2}} \end{array}$$

where $x_{i}$ represents the vector of the *i*th sample in the feature matrix *X*, and $x_{ir}$ represents the value of the *r*th feature in the *i*th sample. The Euclidean distance is not comprehensive to represent the underlying association among features in omics data, as it is more suitable to reflect the absolute differences in values. In this framework, different samples are considered as different vectors to calculate the cosine similarity matrix, and the association is measured by the angular difference between vectors (Xia *et al*. 2015). The cosine similarity is calculated as follows:

(2) $$\begin{array}{c} s(x_{i},x_{j})=\frac{x_{i}\cdot x_{j}}{||x_{i}|{|}_{2}||||x_{i}|{|}_{2}} \end{array}$$

The cosine similarity is more suitable to construct hyperedges than the Euclidean distance, and we demonstrate the efficiency through controlled experiments in the Section 3.

The KNN algorithm (Zhang and Zhou 2007) was utilized to construct the hypergraph structure for each omics data type based on the sample similarity matrix. Specifically, each patient sample is taken as a vertex in the hypergraph, and its *k* nearest neighbors (including itself) are selected and included in the hyperedge centered on that vertex. In this way, the matrix *G* is constructed as the incidence matrix of the hypergraph *H*, defined as:

(3) H(v,e)={1, if v∈e  0, otherwise 

With this extension, δ(v) represents the degree of the vertex *v* is defined as:

(4) $$\begin{array}{c} \delta (v)=\sum_{e\in E}H(v,e) \end{array}$$

The degree of the hyperedge is defined as:

(5) $$\begin{array}{c} \zeta (e)=\sum_{v\in V}H(v,e) \end{array}$$

Furthermore, $D_{e}$ and $D_{v}$ denote the diagonal matrices of edge degree and vertex degree, respectively.

### 2.3 Hypergraph convolution networks

After constructing hypergraph structure for each omics data type, we build HGCN to perform specific learning on the hypergraph. For this purpose, the relationships of the vertices within the hypergraph need to be converted into a matrix as input to the HGCN. The Laplace operator is often used to convert adjacency matrix to Laplace matrix in graph theory (Merris 1994):

(6) $$\begin{array}{c} L_{n}=I-D^{-\frac{1}{2}}AD^{-\frac{1}{2}} \end{array}$$

where *I* is the identity matrix, *D* is the degree of the vertices in the graph, and *A* is the adjacency matrix of the graph.

Likewise, the Laplace matrix of the hypergraph structure can be adjusted as follows:

(7) $$\begin{array}{c} L_{h}=D_{v}^{-\frac{1}{2}}GD_{e}^{-1}G^{T}D_{v}^{-\frac{1}{2}} \end{array}$$

where $D_{v}$ is the degree matrix of the hypergraph vertices, $D_{e}$ is the degree matrix of the hyperedges, and *G* is the incidence matrix of the hypergraph.

The objective of using HGCN is to learn the association between the input data and the real labels. Single-omics feature data and hypergraph data are input to HGCN to perform convolution operators to extract classification evidence (Feng *et al*. 2019). Specifically, the model requires the following two inputs: one is the feature matrix $X\in R^{n\times d}$ for that omics data type, where *n* is the number of samples and *d* is the number of omics features. Another input is the hypergraph Laplacian matrix $L_{h}\in R^{n\times n}$, which serves as a description of the hypergraph structure.

The HGCN is built by multiple convolutional layers and one fully connected layer, the dimension of each convolutional layer is set according to the dimension of the feature matrix *X*, and the output dimension of the fully connected layer is the number of label classes.

The convolution layer is defined as:

(8) $$\begin{array}{c} {\text{HGConv}}^{\left(l+1\right)}=f\left({\text{HGConv}}^{\left(l\right)},L_{h}\right)\\ =\sigma \left(L_{h}\left({\text{HGConv}}^{\left(l\right)}\right)Z^{\left(l\right)}\right) \end{array}$$

where ${\text{HGCconv}}^{\left(l\right)}$ is the output of layer *l* and $Z^{\left(l\right)}$ is the weight matrix of layer *l*. When l=0, ${\text{HGConv}}^{\left(l\right)}=X$. σ(−) is the activation function of this hidden layer, which is set as the LeakyReLU function in this framework to solve the gradient disappearance problem due to neuron failure.

The output $F^{o}$ of HGCN is used as the result of classification evidence extraction for omics data type *o*, where *n* is the number of samples, *b* is the number of label classes and $F^{o}\in R^{n\times b}$.

### 2.4 Trusted multi-omics integrated classifier

Most of the late integration methods are achieved by linear connection, and these methods default to multi-omics data being stable and reliable. However, the heterogeneity of multi-omics data limit the model classification performance. In addition, different sample data are not guaranteed to satisfy the same collecting environment, which also makes the quality uncertainty. Some datasets even collect omics data from different network sources, leading to significant limitations in learning efficiency.

#### 2.4.1 Evidence parameter estimation

The Dirichlet distribution is a multinomial distribution that has been widely used as a classical method for judging discrete sets in many research directions such as multi-agent reinforcement learning, anomaly detection, data clustering, and local information inference (Ferguson 1973). Therefore, the Trusted Multi-Omics integrated classifier (TMO) module of this framework is inspired by the Trusted Multi-view Classification method proposed by Han *et al*. (2022). Evidence results of each omics data type are subjected to confidence and uncertainty estimation. Confidence and uncertainty parameters for different omics are integrated to improve the accuracy and stability of the framework.

First, the Dirichlet distribution parameter matrix $\alpha^{o}$ is constructed based on the evidence results $F^{o}$ of the omics data type *o*. The Dirichlet distribution parameters are used to calculate the integration variables $P^{o}$ and $U^{o}$:

(9) $$\begin{array}{c} \alpha^{o}=F^{o}+1\\ p_{ij}^{o}=\frac{f_{ij}^{o}}{{\sum_{j}\alpha_{ij}}^{o}}\\ u_{i}^{o}=\frac{b}{{\sum_{j}\alpha_{ij}}^{o}} \end{array}$$

where $p_{ij}^{o}$ is the confidence variable of evidence element $f_{ij}{\;}^{o}$ in $F^{o}$, and $U^{o}\in R^{n}$ is the uncertainty variable for the omics data type *o* in the classification task.

Confidence and uncertainty variables are important parameters for trusted multi-omics integration. If there is more reliable evidence for class *k* of omics data type *o*, it will be assigned a higher probability. Correspondingly, the evidence is unreliable and the probability of that class is decreased (Han *et al*. 2022).

#### 2.4.2 Cross-omics information integration

Having completed the parameter estimation of the single-omics evidence, we need to integrate the evidence information to obtain trusted classification results. The Dempster–Shafer theory (DST) is a method of evidential reasoning proposed by Dempster and later expanded and developed by Shafer (1976). Evidence from different views is combined to produce trusted results (Han *et al*. 2022), and the DST has been widely used in information integration. DST (Shafer 1976) is also used in this article to achieve evidence integration between different omics data type:

(10) $$\begin{array}{c} P^{2m\;+\;2}=\frac{P^{2m\;+\;1}U^{2m}\;+\;P^{2m}U^{2m\;+\;1}\;+\;P^{2m\;+\;1}P^{2m}}{1-\sum_{i\;\neq \;j}p_{i}^{2m\;+\;1}p_{j}^{2m}},(m\geq 0)\\ U^{2m\;+\;2}=\frac{U^{2m\;+\;1}U^{2m}}{1-\sum_{i\;\neq \;j}p_{i}^{2m\;+\;1}p_{j}^{2m}},(m\;\geq \;0) \end{array}$$

where $\sum_{i\;\neq \;j}p_{i}^{2m\;+\;1}p_{j}^{2m}$ as a conflict factor to measure the conflicting information of classification results between two omics data type. *m* is set to a value that cannot be less than 0, Specifically, when m=0, the above equations achieve the integration of $P^{0}$, $U^{0}$ (evidence variables of the first omics data type) with $P^{1}$, $U^{1}$ (evidence variables of the second omics data type) to obtain $P^{2}$ and $U^{2}$ as the integration result. In this way, we complete the integration of all omics data types to obtain the final confidence value $P^{2m\;+\;2}$ and uncertainty value $U^{2m\;+\;2}$.

After the integration of all omics data types, the integrated Dirichlet distribution parameter $\alpha^{2m\;+\;2}$ and the predicted result $F^{2m\;+\;2}$ can be derived:

(11) $$\begin{array}{c} f_{ij}^{2m\;+\;2}=\frac{n*p_{ij}^{2m\;+\;2}}{u_{i}^{2m\;+\;2}}\\ \alpha_{ij}^{2m\;+\;2}=f_{ij}^{2m\;+\;2}\;+\;1 \end{array}$$

Finally, we focus on how to train HGCNs using the Dirichlet distribution. Neural networks are usually trained using a cross-entropy loss function.

(12) $$\begin{array}{c} L_{\text{ce}}=-\sum_{j\;=\;1}^{K}y_{ij}\;\text{log}\;\left(p_{ij}\right) \end{array}$$

We need to adjust the cross-entropy loss function with the Dirichlet distribution probability density function. and the cross-entropy loss function ensures that the correct labels have more influence than the other classes, and does not use incorrect classification information to optimize the model parameters. Therefore, this framework defines the target loss value ${\text{Loss}}_{\text{TMO}}$ as the sum of two functions:

(13) $$\begin{array}{c} {\text{Loss}}_{\text{TMO}}={\text{Loss}}_{\text{right}}\;+\;\lambda_{\text{epoch}}{\text{Loss}}_{\text{wrong}}\\ \lambda_{\text{epoch}}=\text{min}(\text{global}\_\text{epoch},\text{annealing}\_\text{step}) \end{array}$$

where ${\text{Loss}}_{\text{right}}$ is the loss function calculated for correct labels, and ${\text{Loss}}_{\text{wrong}}$ is the complement loss function for incorrect classification information. $\lambda_{\text{epoch}}$ as the complement loss weight between (0,1) and changes dynamically depending on the current epoch. Specifically, due to the significant impact of erroneous evidence in the neural network’s initial stages of training, resulting in a substantial influence on the computed ${\text{Loss}}_{\text{wrong}}$, we have introduced $\lambda_{\text{epoch}}$ to prevent the network from excessively focusing on incorrect evidence information during the early phases of training, where global_step represents the current epoch, while annealing_step is a predefined parameter. We have detailed the loss function in the Supplementary Text S2.

Finally, the overall loss function used for training is as follows:

(14) $${\text{Loss}}_{\text{overall}}=\sum_{i=1}^{N}[{\text{Loss}}_{\text{TMO}}(\alpha_{i}^{\text{inter}})\;+\;\sum_{o=1}^{O}({\text{Loss}}_{\text{TMO}}(\alpha_{i}^{o}))]$$

where $\alpha_{i}^{\text{inter}}$ is the Dirichlet distribution parameter obtained by integrating the multi-omics classification evidence.

HyperTMO is an end-to-end model trained to perform accurate disease classification based on cross-omics correlation information.

## 3 Results

### 3.1 Comparison with other existing multi-omics classification methods

#### 3.1.1 Baselines

We compared the classification performance of HyperTMO with the following seven existing representative methods.

- SVM classifier (Noble 2006).
- Random forest (RF) classifier (Shi and Horvath 2006).
- Gradient tree boosting-based classifier implemented in the XGBoost package (XGBoost) (Chen *et al*. 2015).
- Block PLSDA is implemented based on Mixomics R Package (Rohart *et al*. 2017). It is a supervised method for feature selection from multi-omics data and classification of discrete results.
- Block sPLSDA is based on the Mixomics R Package implementation (Rohart *et al*. 2017), which adds a sparse regularization mechanism to Block PLSDA.
- MoGCN is an early integration method based on graph convolutional neural networks (Li *et al*. 2022).
- MOGONET is a late integration method based on GCNs and VCDN (Wang *et al*. 2021).

#### 3.1.2 Performance comparison

All methods were trained with the same preprocessed data, where MoGCN represents the SOTA method for early integration and MOGONET represents the SOTA method for late integration. Table 1 shows the performance comparison results.

**Table 1.** Performance comparison of HyperTMO with other methods on ROSMAP and BRCA dataset.

Methods	ROSMAP			BRCA		
	ACC	F1	AUC	ACC	F1_weighted	F1_macro
SVM	0.770 ± 0.024	0.778 ± 0.016	0.770 ± 0.026	0.729 ± 0.018	0.702 ± 0.015	0.640 ± 0.017
RF	0.726 ± 0.029	0.734 ± 0.021	0.811 ± 0.019	0.754 ± 0.009	0.733 ± 0.010	0.649 ± 0.013
XGBoost	0.760 ± 0.046	0.772 ± 0.045	0.837 ± 0.030	0.781 ± 0.008	0.764 ± 0.010	0.701 ± 0.017
Block PLSDA	0.742 ± 0.024	0.755 ± 0.023	0.830 ± 0.025	0.642 ± 0.009	0.534 ± 0.014	0.369 ± 0.017
Block sPLSDA	0.753 ± 0.033	0.764 ± 0.035	0.838 ± 0.021	0.639 ± 0.008	0.522 ± 0.016	0.351 ± 0.022
MoGCN	0.803 ± 0.059	0.771 ± 0.097	0.806 ± 0.062	0.804 ± 0.039	0.812 ± 0.031	0.761 ± 0.031
MOGONET	0.855 ± 0.040	0.867 ± 0.035	0.855 ± 0.039	0.815 ± 0.036	0.814 ± 0.037	0.796 ± 0.036
HyperTMO (Ours)	0.875 ± 0.033	0.874 ± 0.033	0.900 ± 0.030	0.858 ± 0.023	0.863 ± 0.023	0.841 ± 0.019

We can observe that HyperTMO produced better mean metric than other methods in all classification tasks, demonstrating the excellent learning ability of our framework. Especially, In the ROSMAP dataset, HyperTMO exhibits remarkable improvement compared to other methods, with an average ACC value reaching 0.875. This represents a 2.0% increase over the second-best method, MOGONET. The AUC achieves 0.900, indicating a more uniform and reasonable distribution in correctly predicting positive and negative samples. These results suggest the framework’s potential application value. furthermore, HyperTMO’s performance improvement on the BRCA dataset is even more pronounced, with average ACC, F1-weighted, and F1-macro values surpassing MOGONET by 4.3%, 4.9%, and 4.5%, respectively.

From the experimental results, it is evident that HyperTMO consistently outperforms other methods in all disease prediction tasks, showcasing its outstanding capability in multi-omics disease association learning. Additionally, HyperTMO maintains lower standard deviations in the cross-validation, reflecting its relatively higher stability. Interestingly, both MoGCN and MOGONET are based on GCN, which represent the SOTA methods of early integration and late integration. However, the performance of MOGONET is more efficient than MoGCN, which seems to indicate that the late integration method is superior to the early integration to some extent.

### 3.2 Comparison in noise immunity

**Figure 3.**
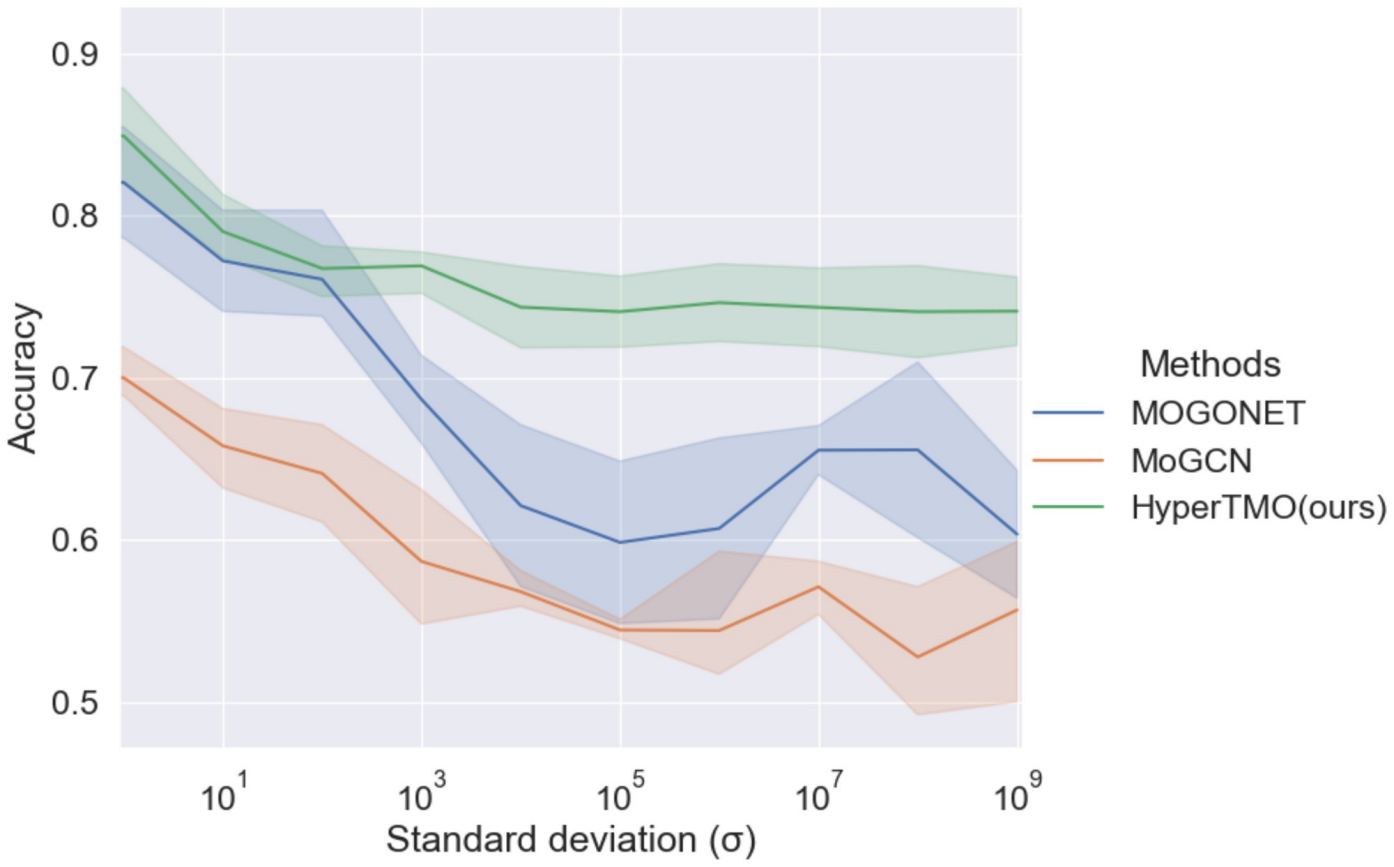
Performance comparison on ROSMAP with different levels of noise.

The noise immunity of the model has extraordinary significance because of the dynamic nature of omics data quality (Burns *et al*. 2022). In order to verify the effectiveness and stability of HyperTMO in the face of noisy data. We add Gaussian noise with different standard deviations (σ) to one of the omics data types and observe the impact of these noises brought on the ACC metrics. The comparison results are shown in Fig. 3.

It can be observed that noise brings learning problems to all the methods and ACC metrics also decreased significantly. However, HyperTMO still outstanding and keeps the highest accuracy. This demonstrates its excellent noise immunity.

### 3.3 Ablation experiments

To further verify the efficiency of the proposed individual modules and theories, we performed ablation experiments to evaluate the efficiency of the hypergraph module and the multi-omics integration module. In this experiment, we first compare HyperTMO with two existing graph structure-based methods and two additional variations of HyperTMO:

- MOGONET: Wang *et al*. proposed a framework where GCN was utilized to perform specificity learning for single-omics data type and construct a cross-omics discovery tensor by VCDN (Wang *et al*. 2021).
- MoGCN: The multi-omics data are fused by SNF methods, and the fusion matrix is input to GCN for training the classifier (Li *et al*. 2022).
- HGCN+VCDN: The HGCN is built for each omics data type to perform feature extraction, and VCDN is used to construct a cross-omics discovery tensor. Moreover, the cross-entropy loss function is used for training.
- GCN+TMO: The GCN is built to perform evidence extraction for each omics data type and is trained to achieve the classification task via a trusted multi-omics integration method.

**Figure 4.**
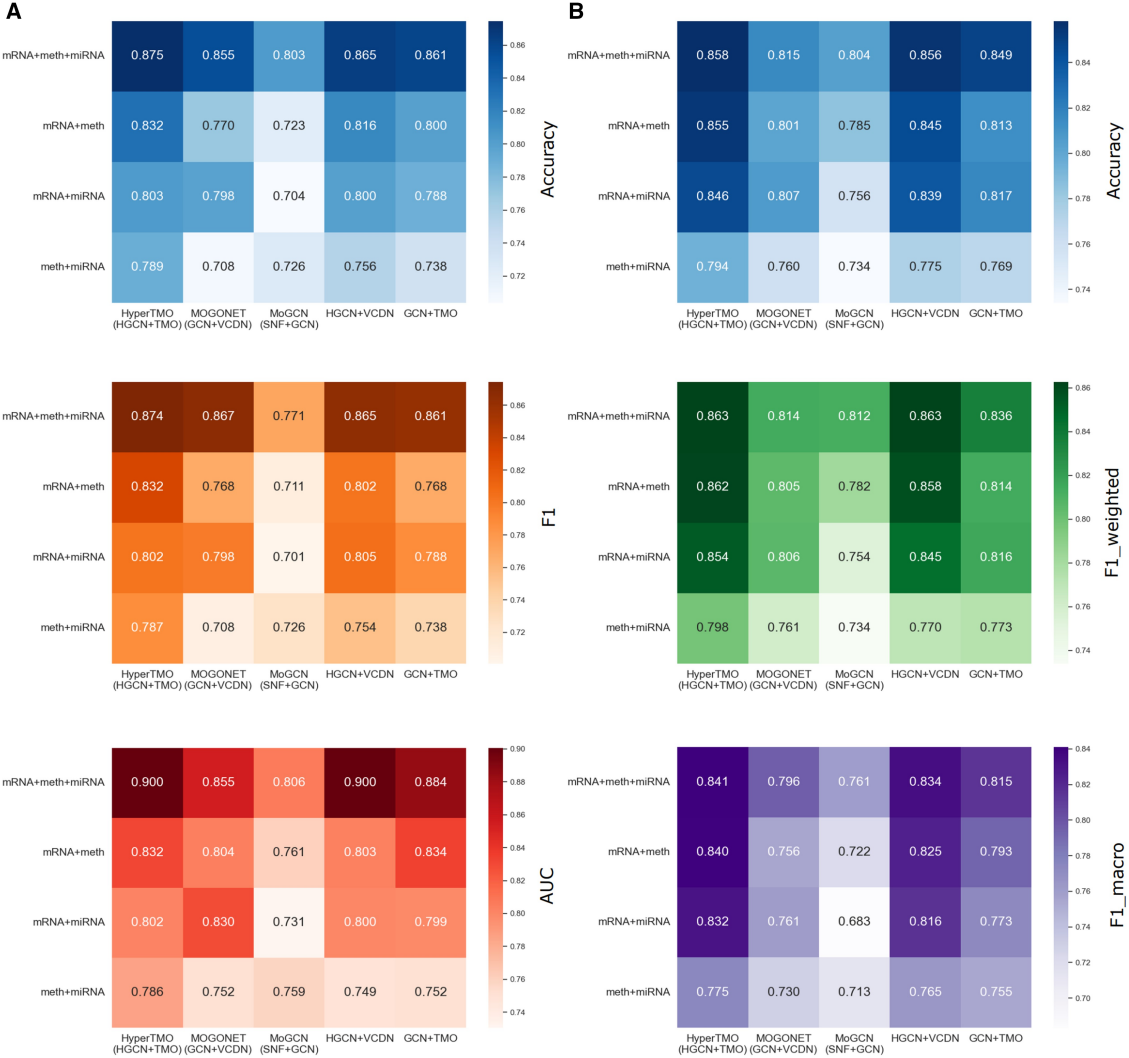
Heatmap of ablation experiments on the hypergraph module and the trusted multi-omics integrated classifier module. (A) Means of evaluation metrics by 5-fold cross-validation on the ROSMAP dataset. (B) Means of evaluation metrics by 5-fold cross-validation on the BRCA dataset.

Heatmaps of the performance comparison results are shown in Fig. 4. Furthermore, we used the cross-entropy loss function to perform single-omics classification tasks based on HGCN. The performance of HyperTMO for two omics data types was also evaluated and the results are shown in Supplementary Fig. S2.

From Fig. 4 and Supplementary Fig. S2, we observe that the trusted multi-omics integration module consistently improves performance and that HyperTMO trained with the three omics data types achieves the best classification results.

In addition, to demonstrate the hypergraph construction method based on the cosine similarity matrix is more suitable for the omics data, we compared it with two representation methods, details are given in Supplementary Text S3.

The hyper-parameter *k* is closely related to the performance of HyperTMO and it represents the number of vertices contained in each hyperedge. We performed controlled experiments with different *k* values to observe the effect on the classification performance. Details of this can be found in Supplementary Text S4.

### 3.4 Biomarker identification based on HyperTMO

The biomarker identification work in this section utilizes the wrapper method. In Supplementary Text S5, we provide a detailed description of the identification process and present the importance rankings of biomarkers across various omics data in the ROSMAP and BRCA datasets. In summary, the multi-omics integration disease prediction framework proposed in this paper provides high-order relationship representation of omics data, deep feature correlations, and reliable feature integration. Compared to other existing multi-omics disease prediction methods, it can uncover more reliable disease-associated biomarkers, imparting significant biological significance to the framework.

## 4 Discussion

To our knowledge, HyperTMO is the first framework to use HGCN and uncertainty estimation to extract evidence information from multi-omics data for patient classification, which enhances the performance on both the single-omics knowledge representation and the multi-omics integrated classification. The experimental results demonstrate that the performance of HyperTMO is far superior to recent multi-omics integration methods, and confirm the excellent noise immunity of our framework.

Although only mRNA, miRNA, and DNA methylation data were used for the experiments in this article, the extensibility of HyperTMO to different omics data types is obvious. For example, Reverse Phase Protein Array data and CNV data can be easily supported by building the corresponding HGCN, while hypergraph construction and trusted multi-omics integration methods can be utilized directly.

In our benchmark analysis, we demonstrate that the classification performance of HyperTMO is much better than other existing methods, while also showing potential for disease detection and risk prediction. However, there are still limitations in HyperTMO. The computation time is increased due to uncertainty estimation and evidence integration, forcing HyperTMO to strike a balance between time efficiency and robust classification performance. In addition, although HyperTMO shows excellent classification performance, we still believe that improvement is possible. In the following, we will discuss the optimization ideas in HyperTMO, which is our future research direction.

Compared to the graph structure, the hypergraph structure we constructed improves the performance of the model on single-omics data. However, the hypergraph structure was only constructed at the beginning of the framework and was used throughout the learning process. This shows the potential for improvement, since the initially constructed hypergraph may not be the most correct representation. Therefore, it is important to evaluate the hypergraph structure dynamically for future research. The model should adaptively adjust the vertices within the hyperedges during training to optimize the hypergraph structure.

Despite deep learning models have achieved great success and are widely used in many applications. However, interpretability is still a common problem for most models (Miotto *et al*. 2018, Zhou *et al*. 2020). Recently, some models have been proposed with interpretability as the primary goal (Elmarakeby *et al*. 2021). These interpretable approaches fuse biological knowledge with neural network structures, and classification results can be interpreted at the biological level through connections between network layers. Most of these models are based on multilayer perceptron or DNN, but it is difficult to apply to Graph Neural Network (GNN), which is commonly utilized for integrated multi-omics classification. Future research should improve the biological interpretability of GNN, which will facilitate the development of biological research and biomedical applications (Shao *et al*. 2021). Therefore, the direction of our future work is to optimize the hypergraph structure during training and to investigate it in terms of interpretability.

## 5 Conclusion

In this article, we propose HyperTMO, a framework for trusted multi-omics integrated disease classification. We construct hypergraph structures for omics data for the first time, which accurately represent the underlying multidirectional relationships in the omics data compared with graph structures. HGCNs are built for hypergraph-specific learning to extract classification evidence for each omics data type. Finally, evidence information was integrated through a trusted multi-omics integration method. The cross-omics complementary relationships are efficiently exploited to solve the accuracy, generalizability, and robustness challenges of the model due to the heterogeneity of multi-omics data.

## Supplementary Material

btae159_Supplementary_Data
