# Supplementary material for: HyperTMO: a trusted multi-omics integration framework based on hypergraph convolutional network for patient classification
Source: Bioinformatics. 2024 Mar 26;40(4):btae159. doi: 10.1093/bioinformatics/btae159 (PMC11212491; doi:10.1093/bioinformatics/btae159)
Supplement: btae159_Supplementary_Data [file btae159_supplementary_data.zip › Supplemental_Text.pdf]

Supplementary: Text

S1: Data preprocessing

It is necessary to compare fairly the performance of HyperTMO with baseline models. Therefore, we performed the preprocessing method from Wang et al. in MOGONET to remove artifacts and redundant features in each omics data, and MOGONET was compared as a SOTA model in the experimental part [1] . Specifically, all omics features with no signal or low variance values were first filtered out (0.1 for mRNA expression data and 0.001 for DNA methylation data). The variance queue of the miRNA expression data was set to 0 because of the small number of miRNA features. Second, we evaluated whether the features were universal under different labels by calculating the ANOVA F-value to pre-screen the features, and the number of preselected features is shown in Table S1.

Table S1: Summary of datasets.

ROSMAP				BRCA			
Number of samples		Number of features		Number of samples		Number of features	
NC	169	mRNA	200	Normal-like	115	mRNA	1000
AD	182	Meth	200	basal-like	131	meth	1000
		miRNA	200	HER2	46	miRNA	503
				Luminal A	436		
				Luminal B	147		

S2: Loss function of HyperTMO

We adjusted the cross-entropy loss function with the dirichlet distribution probability density function, and the dirichlet distribution is defined as:

$$\mathrm{Dir}(X \mid \alpha) = \frac{1}{B(\alpha)} \prod_{k=1}^{K+1} X_k^{\alpha_k - 1} \tag{1}$$

the adjusted loss function is defined as:

$$\begin{aligned} Loss_{right}(\alpha_i) &= \int \left[\sum_{j=1}^{K} - y_{ij} log\, g\left(p_{ij}\right) \right] D(P_i \mid \alpha_i) dP_i \\ &= \sum_{j=1}^{K} y_{ij} \Psi \left(\sum_{j=1}^{K} \alpha_{ij} \right) - \Psi\left(\alpha_{ij}\right) \end{aligned} \tag{2}$$

where Ψ (x) is the digamma function:

$$\begin{aligned} \Psi(x) &= \frac{\Gamma'(x)}{\Gamma(x)} \\ \Gamma(x) &= \int_0^{+\infty} t^{x-1} e^{-t} dt \end{aligned} \tag{3}$$

The cross-entropy loss function ensures that the correct labels have more influence than the other classes, and does not use incorrect classification information to optimize the model parameters. Therefore, this framework defines the target loss value $Loss_{TMO}$ as the sum of two functions:

$$Loss_{TMO} = Loss_{right} + \lambda_{epoch} Loss_{wrong}$$

where $Loss_{right}$ is the loss function calculated for correct labels, and $Loss_{wrong}$ is the complement loss function for incorrect classification information and we will introduce it right away. λ_{epoch} as the complement loss weight between (0,1) and changes dynamically depending on the current epoch.

We introduce the KL divergence term to define the $Loss_{wrong}$ as:

$$Loss_{wrong}(\alpha_i) = KL\left[Dir\left(\mathbf{p}_i \mid \tilde{\boldsymbol{\alpha}}_i\right) \parallel Dir(\mathbf{p}_i \mid \mathbf{1})\right]$$

$$= \sum_{k=1}^{K} \left(\tilde{\boldsymbol{\alpha}}_{ik} - 1\right) \left[\Psi\left(\tilde{\boldsymbol{\alpha}}_{ik}\right) - \Psi\left(\sum_{k=1}^{K} \tilde{\boldsymbol{\alpha}}_{ik} \right) \right] + \log\left(\Gamma\left(\sum_{k=1}^{K} \tilde{\boldsymbol{\alpha}}_{ik} \right) \right) - \sum_{k=1}^{K} \left(\log\left(\Gamma\left(\tilde{\boldsymbol{\alpha}}_{ik}\right)\right) - \log\left(\Gamma(K)\right)\right) \tag{4}$$

where $\tilde{\boldsymbol{\alpha}}_i = (1 - \alpha_i) y_i + \alpha_i$ is the adjusted parameter that takes into account the incorrect classification information [2] .

Finally, the overall loss function used for training is as follows:

$$Loss_{overall} = \sum_{i=1}^{N} \left[Loss_{TMO}\left(\alpha_i^{inter}\right) + \sum_{o=1}^{O} \left(Loss_{TMO}\left(\alpha_i^{o}\right) \right) \right] \tag{5}$$

where α_i^{inter} is the dirichlet distribution parameter obtained by integrating the multi-omics classification evidence.

S3: Ablation experiments of HyperTMO

To further verify the efficiency of the proposed individual modules and theories, we performed ablation experiments to evaluate the efficiency of the multi-omics integration classifier module and the hypergraph convolution networks module.

In this ablation experiment, we first compare HyperTMO with two existing graph structure-based methods and two additional variations of HyperTMO.

- MOGONET: Wang et al. proposed a framework where GCN was utilized to perform specificity learning for single-omics data type and construct a cross-omics discovery tensor by VCDN. Moreover, the cross-entropy loss function was used for training [1] .
- MoGCN: The multi-omics data are fused by SNF methods, and the fusion matrix is input to GCN for training the classifier [3] .
- HGCN+VCDN: The HGCN is built for each omics data type to perform feature extraction, and VCDN is used to construct a cross-omics discovery tensor. Moreover, the cross-entropy loss function is used for training.
- GCN+TMO: The GCN is built to perform evidence extraction for each omics data type and is trained to achieve the classification task via a trusted multi-omics integration method.

Heatmaps of the performance comparison results are shown in Figure S1. Furthermore, we used the cross-entropy loss function to perform single-omics classification tasks based on HGCN. The performance of HyperTMO for two omics data types was also evaluated and the results are shown in Figure S2.

From Figure S1 and Figure S2, we observe that the trusted multi-omics integration module consistently improves performance and that HyperTMO trained with the three omics data types achieves the best classification results.

In addition, to demonstrate the hypergraph construction method based on the cosine similarity matrix is more suitable for the omics data, we compared it with two representation methods:

- Graph: the method proposed by Wang et al. to represent the graph structure for omics data [1] .
- Hypergraph_euclidean: Euclidean distance was utilized to discover the similarity between samples and construct the hypergraph structure by KNN algorithm.

The results for each omics data type are shown in Table S2. From Figure S1 and Table S2, we observe that HGCN can significantly improve the classification performance, and the hypergraph construction based on cosine similarity can analyze the potential relationships of the omics data more accurately, leading to further improvement of the classification performance.

Table S2: Performance comparison of graph methods and hypergraph methods based on cosine similarity or Euclidean distance for each omics data type.

Omics	Methods	ROSMAP		
		ACC	F1	AUC
mRNA	Graph	0.7434 ± 0.0254	0.7433 ± 0.0351	0.7879 ± 0.0432
	Hypergraph_euclidean	0.7641 ± 0.0403	0.7913 ± 0.0468	0.8041 ± 0.0608
	Hypergraph_cosine(ours)	0.7893 ± 0.0365	0.8064 ± 0.0268	0.8064 ± 0.0268
meth	Graph	0.6094 ± 0.0443	0.6108 ± 0.0433	0.7539 ± 0.0482
	Hypergraph_euclidean	0.6485 ± 0.0484	0.6471 ± 0.0652	0.6462 ± 0.0638
	Hypergraph_cosine(ours)	0.7093 ± 0.0600	0.7632 ± 0.0451	0.7621 ± 0.0453
miRNA	Graph	0.6396 ± 0.0469	0.6395 ± 0.0435	0.7371 ± 0.0212
	Hypergraph_euclidean	0.6571 ± 0.0756	0.6597 ± 0.0436	0.7806 ± 0.0326
	Hypergraph_cosine(ours)	0.6468 ± 0.0685	0.6560 ± 0.0357	0.7894 ± 0.0355

Omics	Methods	BRCA		
		ACC	F1-weighted	F1-macro
mRNA	Graph	0.7939 ± 0.0318	0.7942 ± 0.0315	0.7619 ± 0.0196
	Hypergraph_euclidean	0.8419 ± 0.0368	0.8599 ± 0.0248	0.8278 ± 0.0462
	Hypergraph_cosine(ours)	0.8411 ± 0.0203	0.8574 ± 0.0217	0.8370 ± 0.0163
meth	Graph	0.6958 ± 0.0341	0.6944 ± 0.0243	0.6435 ± 0.0338
	Hypergraph_euclidean	0.7658 ± 0.0430	0.7889 ± 0.0404	0.7616 ± 0.0378
	Hypergraph_cosine(ours)	0.7600 ± 0.0220	0.7730 ± 0.0197	0.7504 ± 0.0137
miRNA	Graph	0.7087 ± 0.0194	0.7087 ± 0.0193	0.6607 ± 0.0135
	Hypergraph_euclidean	0.7278 ± 0.0361	0.7378 ± 0.0340	0.7184 ± 0.0427
	Hypergraph_cosine(ours)	0.7943 ± 0.0081	0.7993 ± 0.0040	0.7555 ± 0.0150

S4: Performance of HyperTMO under different hyper-parameters k

The hyper-parameter k is closely related to the performance of HyperTMO and is one of the most important hyperparameters. It represents the number of vertices contained in each hyperedge and must be greater than or equal to 2 because we set the hyperedge must include self-connections in our experiments. We performed controlled experiments with different k values to observe the effect on the classification performance. The mean metric results are shown in Figure S3.

We observe that the best performance is achieved in the ROSMAP and BRCA datasets when k is 3 and 4, respectively. It is worth mentioning that when k is 2, the framework constructs graph structures to learn while the performance is still better than other baseline models, which is more evidence of the effectiveness of our representation method and the multi-omics integration method.

S5: Biomarker Identification Based on the HyperTMO Framework

Currently, biomarker research employing machine learning algorithms predominantly employs a wrapper method. The wrapper method, based on a given disease prediction framework, identifies biomarkers by observing how controlling the expression of omics features influences the framework's predictive outcomes. Due to its comprehensive feature analysis and better scalability, the wrapper method has been widely applied by researchers for many years [4] .

The biomarker identification work in this section also utilizes the wrapper method. Initially, the best-performing models are obtained through extensive training on the original dataset. Then, individual feature values within each omics dataset are controlled, i.e., each feature is set to zero, and the hypergraph structure is reconstructed. This reconstructed data is input into the saved model for metric computation. The importance of features is ranked by observing the extent to which it affects the F1 score in classification metrics. Features with high rankings are considered as key biomarkers associated with the relevant diseases.

The multi-omics integration disease prediction framework proposed in this paper provides high-order relationship representation of omics data, deep feature correlations, and reliable feature integration. Compared to other existing multi-omics disease prediction methods, it can uncover more reliable disease-associated biomarkers, imparting significant biological significance to the framework.

We present the importance rankings of biomarkers in various omics data on the ROSMAP and BRCA datasets. The top 10 ranked biomarkers are shown in Table S3 and S4 with those biomarkers previously validated in related literature highlighted in bold.

Table S3: Biomarkers identified on ROSMAP dataset based on wrapped approach

meth	mRNA	miRNA
**CCL3**	**NPNT**	**hsa-miR-425-3p**
**AGA**	**CDK18**	**hsa-miR-33a**
**TMC4**	**KIF5A**	**hsa-miR-491-5p**
**TTC15**	**SPACA6**	hsa-miR-640
**ATP6V1A**	**ARRDC2**	**hsa-miR-362-3p**
**HYAL2**	APLN	**hsa-miR-127-3p**
**CDH1**	**TCEA3**	**hsa-miR-106a**
AGMAT	SCD	**hsa-miR-206**
**LRRC39**	SYTL1	hsa-miR-577
GPR25	**NRIP2**	**hsa-miR-651**

In Table S3, many of the biomarkers have been found to have latent associations with symptoms or complications related to Alzheimer's disease. For instance, Marciniak et al. demonstrated in 2015 through experiments on mice that the epigenomic biomarker CCL3 acts as a hippocampal neuroregulator, influencing synaptic plasticity mechanisms involved in learning and memory functions [5] . CCL3 stands out as the most important methylation biomarker for Alzheimer's disease in our method. Furthermore, The Chinese Academy of Sciences has revealed that NPNT can play a role in maintaining the homeostasis of muscle cells. When there is an abnormality in the NPNT regulatory pathway, it can lead to an increased susceptibility of skeletal muscle cells to

undergo aging, which may serve as a potential trigger for symptoms related to Alzheimer's disease [6] . NPNT is identified as the most crucial mRNA biomarker derived from the analysis based on the HyperTMO presented in our experiment.

Similarly, in Table S4, Chen et al. conducted a statistical study and found that SLC6A15 is a sodium-dependent neutral amino acid transporter that exhibits dysregulated expression in breast cancer patients. It is considered a potential drug target for cancer therapy [7] . SLC6A15 is identified as the most important mRNA biomarker uncovered by our framework using a wrapper method. In a 2017 study, luciferase assays were used to confirm the association of hsa-mir-204 with breast cancer symptoms. The results indicated that overexpression of hsa-mir-204 inhibited normal cell proliferation and migration processes, promoting invasion and apoptosis in some cells. This suggests that hsa-mir-204 could serve as a suppressor in breast cancer and be a targeted research focus [8] . This biomarker is also recognized as the most critical miRNA biomarker in Table S4.

While a small portion of the biomarkers in the tables has not yet been validated by the academic community, there is reason to believe that novel biomarkers identified through artificial intelligence methods have the potential to contribute to disease diagnosis and prognosis, furthering the development of human life sciences.

Table S4: Biomarkers identified on BRCA dataset based on wrapped approach

meth	mRNA	miRNA
**GPR37L1**	**SLC6A15**	**hsa-mir-204**
**MT1DP**	**SLC6A14**	**hsa-mir-135b**
KRTAP3-3	**SOX11**	**hsa-mir-205**
FLJ41941	**AMY1A**	**hsa-mir-223**
**MIR645**	CCDC150	hsa-mir-1269
**MIR563**	**MDGA2**	**hsa-let-7c**
ATP10B	**ANKRD45**	hsa-mir-934
**OR1J4**	GABRA5	**hsa-mir-18a**
TMEM207	**EN1**	**hsa-mir-502**
**TAL2**	**ZIC1**	hsa-mir-505

Supplementary: Figures

Figure S1: Heatmap of ablation experiments on the hypergraph module and the trusted multi-omics integrated classifier module.

(A) Means of evaluation metrics by 5-fold cross validation on the ROSMAP dataset.

(B) Means of evaluation metrics by 5-fold cross validation on the BRCA dataset.

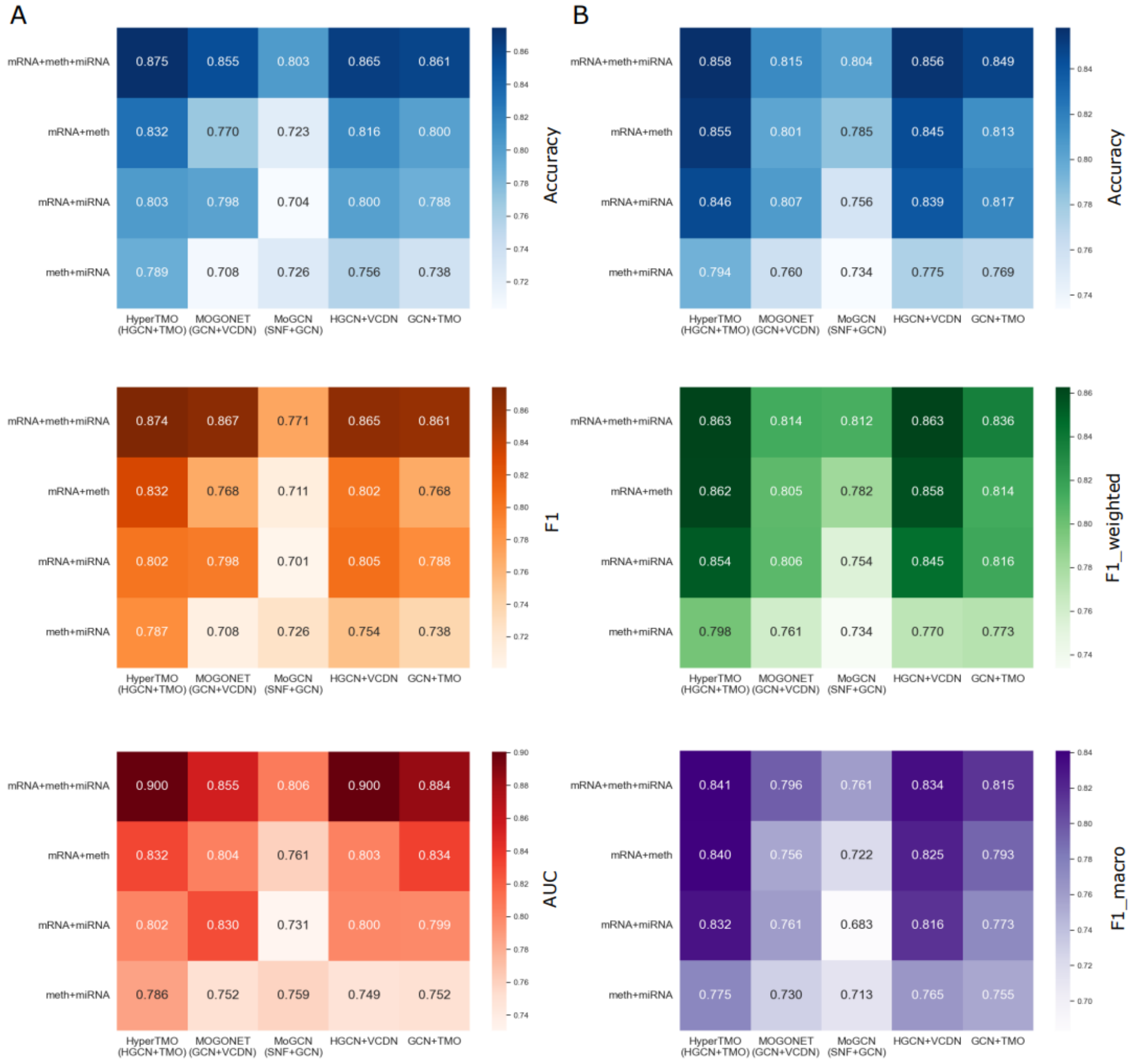

Figure S2: Performance comparison of multi-omics classification via HyperTMO and single-omics classification via HGCN. Means of evaluation metrics with standard deviations by 5-fold cross validation are shown in the figure.

(A) Results of the binary classification task on the ROSMAP dataset.

(B) Results of the multi-class classification task on the BRCA dataset.

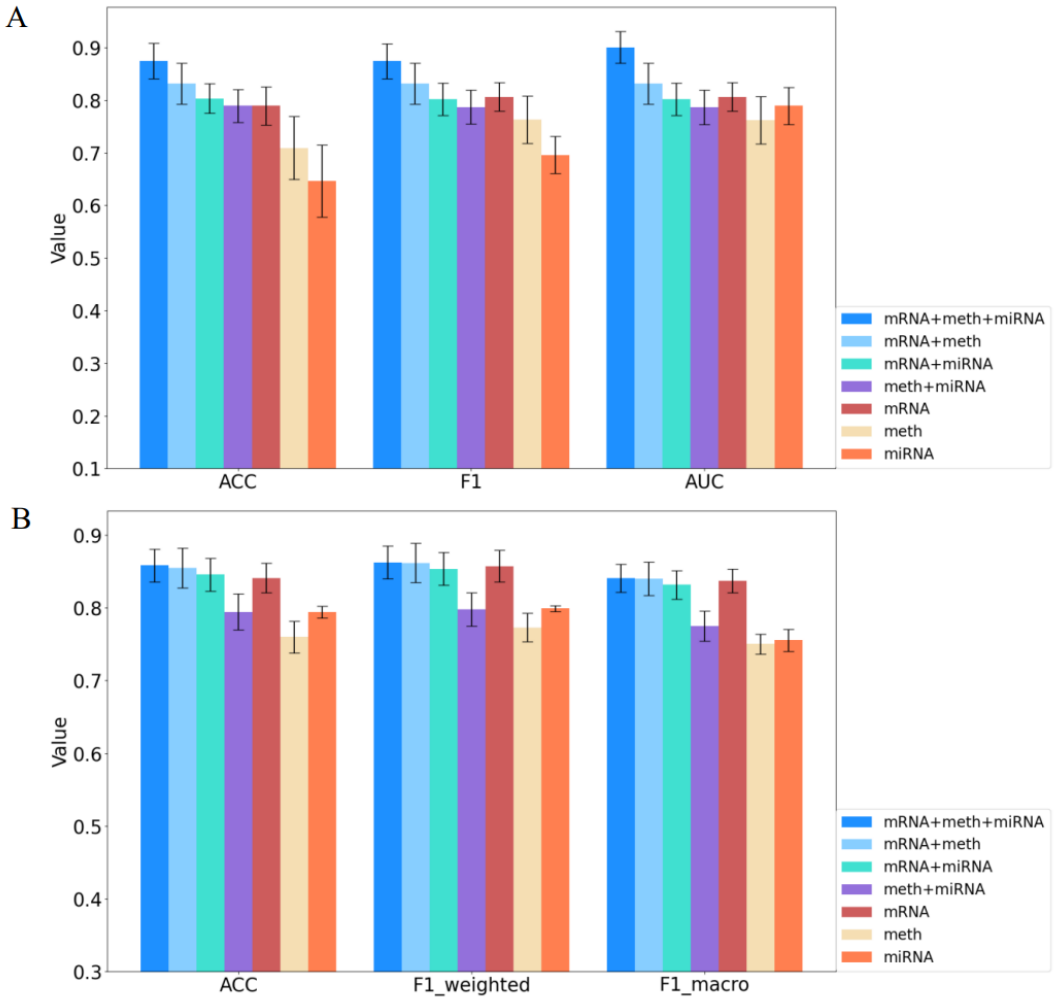

Figure S3: Performance of HyperTMO under different hyper-parameters k.

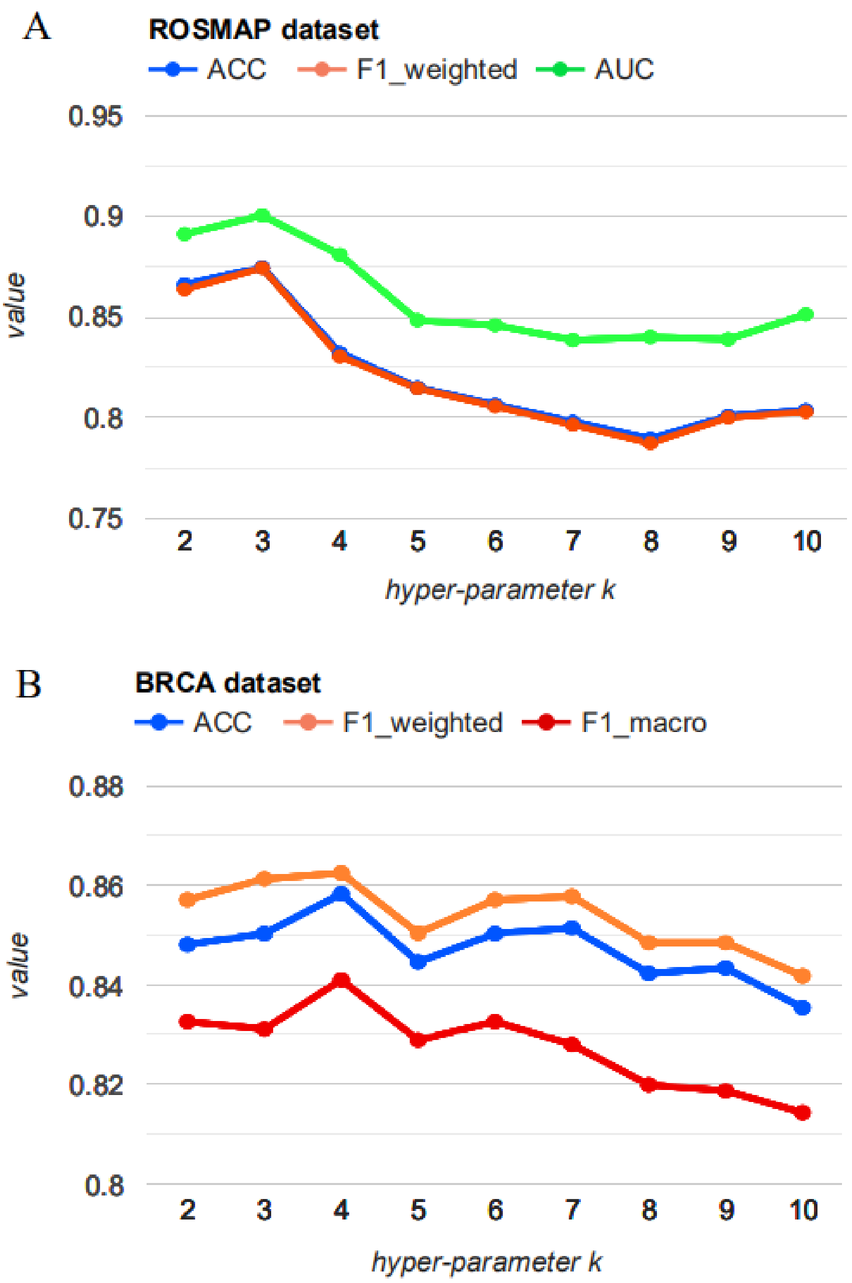

Bibliography

[1] Wang T, Shao W, Huang Z, et al. MOGONET integrates multi-omics data using graph convolutional networks allowing patient classification and biomarker identification[J]. Nature communications, 2021, 12(1): 3445.

[2] Han Z, Zhang C, Fu H, et al. Trusted multi-view classification[J]. arXiv preprint arXiv:2102.02051, 2021.

[3] Li X, Ma J, Leng L, et al. MoGCN: A multi-omics integration method based on graph convolutional network for cancer subtype analysis[J]. Frontiers in Genetics, 2022, 13: 127.

[4] Li Y, Mansmann U, Du S, et al. Benchmark study of feature selection strategies for multi-omics data[J]. BMC bioinformatics, 2022, 23(1): 1-18.

[5] Marciniak E, Faivre E, Dutar P, et al. The Chemokine MIP-1α/CCL3 impairs mouse hippocampal synaptic transmission, plasticity and memory[J]. Scientific reports, 2015, 5(1): 1-11.

[6] Wu Z, Lu M, Liu D, et al. m6A epitranscriptomic regulation of tissue homeostasis during primate aging[J]. Nature Aging, 2023: 1-17.

[7] Chen Y, Li H, Liang W, et al. SLC6A15 acts as a tumor suppressor to inhibit migration and invasion in human papillary thyroid cancer[J]. Journal of Cellular Biochemistry, 2021, 122(8): 814-826.

[8] Shen S Q, Huang L S, Xiao X L, et al. miR-204 regulates the biological behavior of breast cancer MCF-7 cells by directly targeting FOXA1[J]. Oncology reports, 2017, 38(1): 368-376.
