# Supplementary figures and images for: HyperTMO: a trusted multi-omics integration framework based on hypergraph convolutional network for patient classification
Source: Bioinformatics. 2024 Mar 26;40(4):btae159. doi: 10.1093/bioinformatics/btae159 (PMC11212491; doi:10.1093/bioinformatics/btae159)

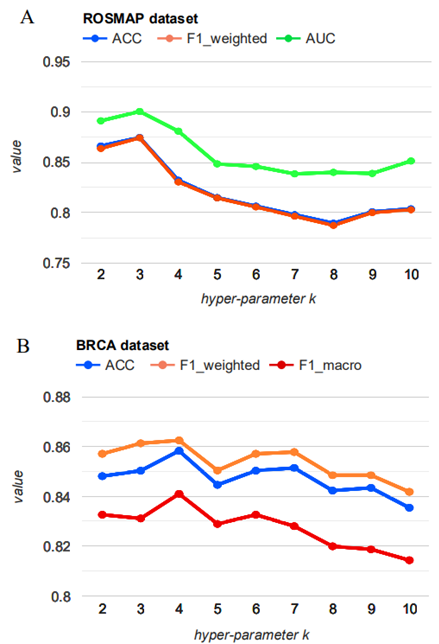

Supplement: btae159_Supplementary_Data [file btae159_supplementary_data.zip › figs3.png]

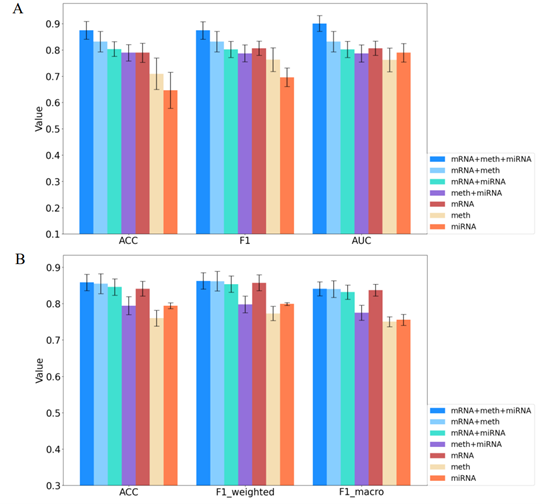

Supplement: btae159_Supplementary_Data [file btae159_supplementary_data.zip › figs2.png]

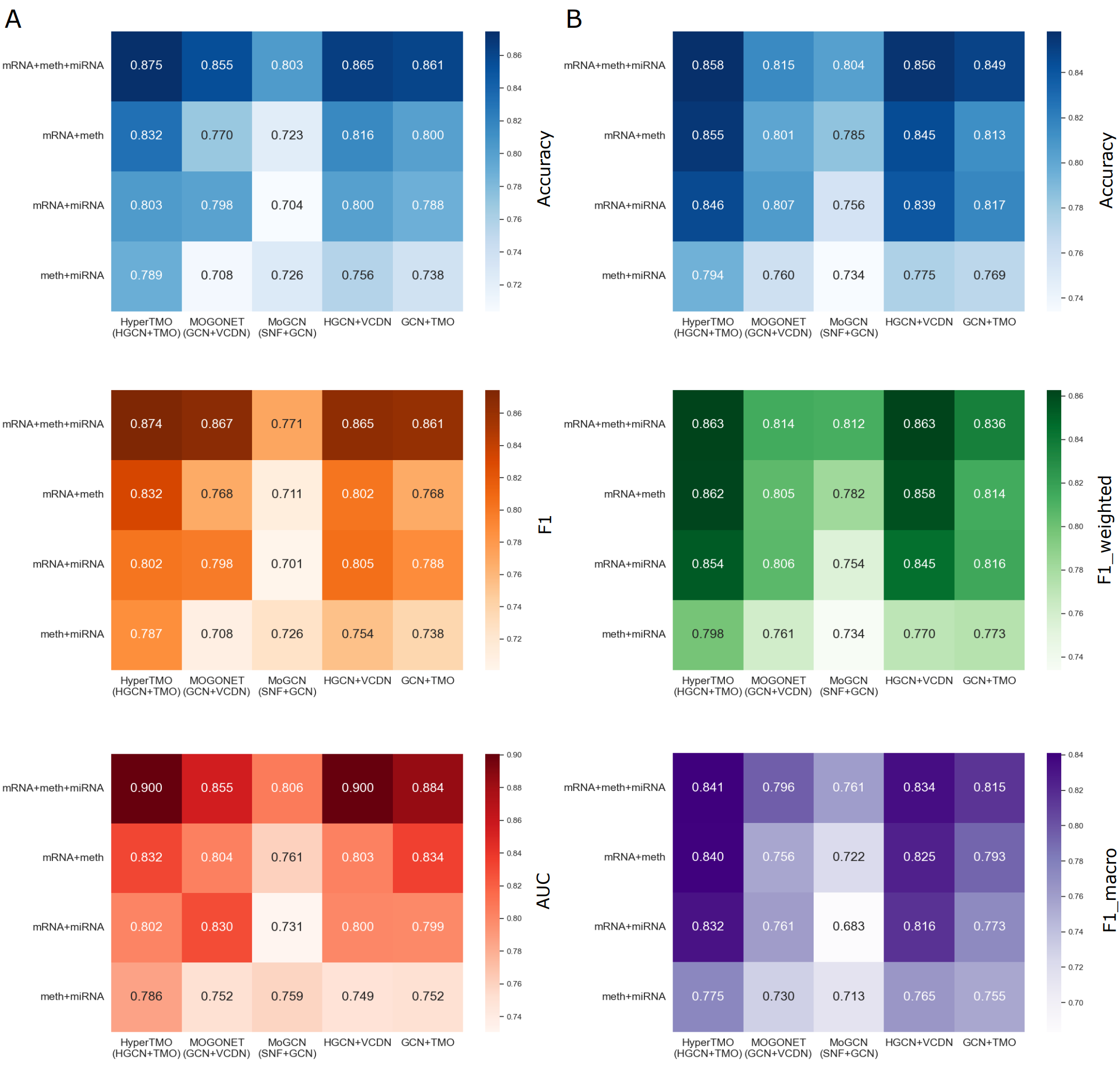

Supplement: btae159_Supplementary_Data [file btae159_supplementary_data.zip › figs1.png]
